# A comparison of the expression patterns and diagnostic capability of the ncRNAs NEAT1 and miR-34a in non-obstructive azoospermia and severe oligospermia

**DOI:** 10.1186/s40246-025-00742-9

**Published:** 2025-03-31

**Authors:** Aya Salman, Abdullah F. Radwan, Olfat G. Shaker, Adel A., Ghadir A. Sayed

**Affiliations:** 1 Department of Biochemistry & Molecular Biology, Faculty of Pharmacy, Egyptian Russian University, Cairo, 11829 Egypt; 2 Department of Pharmacy, Kut University College, Wasit, 52001 Iraq; 3 Department of Medical Biochemistry and Molecular Biology, Kasr AlAiny Faculty of Medicine, Cairo University, Cairo, 12613 Egypt; 4 Department of Andrology, Sexology, and STIs, Faculty of Medicine, Cairo University, Cairo, 12613 Egypt

**Keywords:** Diagnosis, Infertility, miR-34a, NEAT1, Non-obstructive azoospermia, Severe oligospermia, Transcriptomics

## Abstract

Infertility is a major global health problem, affecting 8–12% of couples worldwide, with male causes contributing to approximately 50% of cases. Notably, around 15% of infertile men are azoospermic. Consequently, there is a critical necessity to find noninvasive biomarkers to help in diagnosing and assessing the susceptibility of patients with various infertility disorders. This study is designed to determine the roles of NEAT1 and miR-34a as diagnostic and susceptibility biomarkers for non-obstructive azoospermia and severe oligospermia. The interactions between these non-coding RNA (ncRNAs) were explored, along with their correlations to hormonal profiles and clinical parameters like sperm count and motility. The potential of serum NEAT1 and miR-34a as diagnostic biomarkers for these conditions was explored. The study included 100 participants: 40 non-obstructive azoospermia patients, 40 severe oligospermia patients, and 20 healthy controls. Quantitative real-time PCR and transcriptomics-based bioinformatics tools were employed to explore the co-expression networks and molecular interactions of NEAT1, miR-34a, SIRT1, and their associated hormonal and genetic pathways. Results indicated that NEAT1 was significantly downregulated in severe oligospermia patients, while its levels in non-obstructive azoospermia patients did not differ significantly from healthy controls. Furthermore, serum miR-34a expression was considerably upregulated in both patient groups compared to controls. This study highlights the promise of serum NEAT1 and miR-34a as diagnostic markers for non-obstructive azoospermia and severe oligospermia. These findings provide valuable insights into male infertility and indicate potential avenues for personalized treatment strategies.

**Supplementary Information:**

The online version contains supplementary material available at 10.1186/s40246-025-00742-9.

## Graphical Abstract

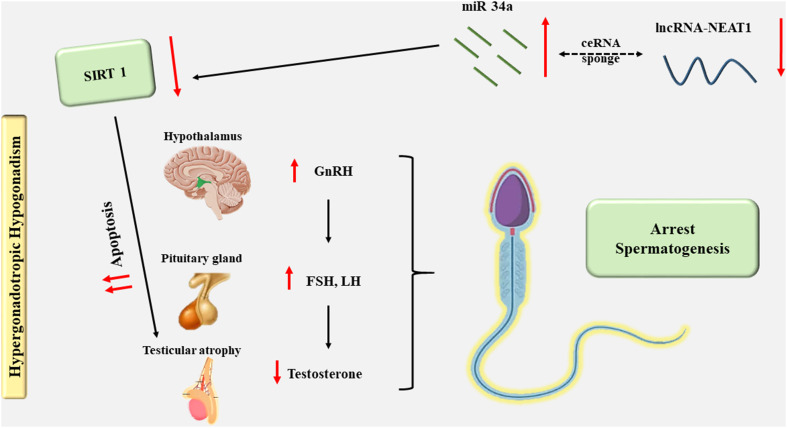

## Introduction

Infertility is a major global health concern, impacting 8–12% of couples worldwide. In about half of these cases, male factors are the primary cause, with 15% of infertile men being azoospermic [1, 2]. Male infertility is a complex medical issue resulting from various pathological conditions [3]. Its defining feature is the inability to produce enough sperm to fertilize an egg [4]. The prevalence of male infertility is rising to alarming levels worldwide, and it has a variety of negative effects on couples. Male infertility is primarily caused by genetics [5]. Various regulatory processes, such as RNA methylation and noncoding RNA (ncRNA), play crucial roles in the formation of healthy sperm throughout the intricate stages of spermatogenesis. Both mechanisms are essential for normal sperm development [6].

ncRNAs are essential in the genetic regulation involved in azoospermia. They are categorized based on their length: long ncRNAs (lncRNAs), which exceed 200 nucleotides, and small or short ncRNAs (sncRNAs), which are under 200 nucleotides [7]. Within the sncRNA group, microRNAs (miRNAs) are prominent regulators of gene expression across various cellular pathways and have been extensively researched in the context of the reproductive system [8, 9].

The function of miRNAs in the posttranscriptional control of spermatogenesis has drawn increasing amounts of attention. The miR-34 family, particularly miR-34a, has been shown to have a significant role in spermatogenesis. miR-34a, which is predominantly expressed in the testes, is situated at the chromosomal location 1p36.22 [10, 11]. This microRNA is implicated in various conditions, including cancers and cardiovascular diseases and regulates genes associated with essential biological processes such as cell proliferation, survival, apoptosis, migration, invasion, and angiogenesis, underscoring its role in cancer development. Additionally, miR-34a serves as a potential biomarker for identifying atherosclerosis, a leading contributor to cardiovascular disease [12–14]. Research indicates that the miR-34 family is significantly involved in the processes of spermatogenesis and the motility of sperm.

SIRT1 is a NAD+-dependent protein deacetylase that has a role in controlling gene expression and cellular events, stress response, and inflammation [15]. It has various functions including aging, neuroprotection, oncogenesis, and fertility because it’s involved in regulating physiological and pathological processes of cellular homeostasis and genome stability [16]. MiR-34a inhibition and SIRT1 activation may be used as treatment options for diabetes mellitus-induced male infertility because the miR-34a/SIRT1 was found to regulate testicular apoptotic cell death [17]. While miR-34a has been shown to inhibit cancer development and contribute to cardiovascular diseases, aging, and hearing loss [18, 19], owing to its pro-apoptotic role, its specific role in the testis remains largely unexplored.

LncRNAs are involved in numerous diseases and biological functions by influencing gene expression through various mechanisms [20–22]. In a study conducted in 2017, researchers identified 1,534 lncRNAs that were differentially expressed between round sperm cells and pachytene spermatocytes, as well as 1,630 lncRNAs in spermatogonia [23]. These lncRNAs significantly regulate spermatogenesis in mammals, acting either cis or trans on target genes. Their expression levels and tissue specificity vary at different stages of spermatogenesis, highlighting their role as guiding molecules in specific signaling pathways [24, 25]. According to previous studies, one of the mechanisms lncRNAs can regulate the expression of target genes is by serving as “sponges” for miRNAs [26, 27]. The competitive endogenous RNA (ceRNA) process is involved in this molecular mechanism. A previous study revealed that lncRNAs can influence spermatogenesis by binding to miRNAs [28].

In 2007, NEAT1 was identified as a lncRNA that is predominantly found in the nucleus and localized in paraspeckles [29]. Transcribed by RNA polymerase II from the chromosome 11q13.1 region associated with multiple endocrine neoplasia type 1 [30]. NEAT1 regulates the expression of lactate dehydrogenase A (LDHA), an enzyme critical for glycolysis, by functioning as a ceRNA for miR-34a. Understanding the roles of lactate and relevant molecular mechanisms in spermatogenesis relies on the specific deletion of either LDHA or lactate dehydrogenase B (LDHB) [31, 32]. Through its ceRNA activity, NEAT1 impacts cell survival and invasion by modulating the Wnt/β-catenin signaling pathway and inhibiting the miR-34a/SIRT1 axis [33].

This study aimed to compare the serum levels of NEAT1 and miR-34a in men with infertility, specifically those with non-obstructive azoospermia or severe oligospermia, against those of fertile men. There is a growing interest in clinical approaches that utilize less invasive, cost-effective tools and biomarkers for screening and predicting male infertility.

## Methodology

### Study sample

This study was conducted using blood serum samples from Egyptian men referred to El-Kasr Al-Ainy Hospital at Cairo University of Medical Sciences between April 2024 and September 2024. All participants provided informed consent. The study included two groups of infertile men: those with non-obstructive azoospermia (*n* = 40) and those with severe oligospermia (*n* = 40). Non-obstructive azoospermia was defined as the absence of sperm in two consecutive semen analyses, while severe oligospermia was defined as a sperm count of fewer than 5 million sperm/ml, based on the latest WHO guidelines for semen analysis published in 2021 [34, 35]. Non-obstructive azoospermia was confirmed in participants by the absence of sperm in at least two separate semen analyses, along with normal results for post-ejaculation urine analysis to rule out retrograde ejaculation. Additionally, diagnostic evaluations such as testicular ultrasound and hormonal assessments were conducted to exclude obstructive causes and confirm the diagnosis of non-obstructive azoospermia. Participants underwent thorough clinical assessments, including medical and surgical histories, hormonal evaluations, genetic testing for sex chromosome abnormalities, semen analysis, and ultrasound examination. None of the participants or their partners had used contraceptives, and their partners had not conceived within the past year with no signs of female infertility. Exclusion criteria included obstructive azoospermia, teratozoospermia, mild or moderate oligospermia, alcohol use, endocrine disorders, or medication use. Obstructive azoospermia was diagnosed through clinical and physical examinations to identify any signs of obstruction, such as a history of infections, surgeries, or trauma affecting the reproductive tract. Imaging studies, including scrotal and transrectal ultrasounds, were performed to detect any structural abnormalities or blockages in the testes, epididymis, vas deferens, seminal vesicles, or prostate. Additionally, semen analysis confirmed the absence of sperm in the semen sample, while normal testicular function was verified through hormonal testing. Men with conditions such as cancer, diabetes, autoimmune disorders, gastrointestinal issues, or lung or kidney diseases were also excluded. Twenty healthy men with normal semen parameters according to WHO standards [35] were selected as the control group. Sperm analysis data were obtained from patient records to examine sperm characteristics.

### Sample size calculation

To determine the optimal sample size for evaluating the targeted biomarkers, including NEAT1, miR-34a, and serum hormones such as LH, FSH, prolactin, testosterone, and E2, the statistical tool G*Power was utilized. This calculation was based on several pivotal parameters: the effect size, reflecting the magnitude of differences in biomarker expression levels between the patient and control groups; the type I error rate (α), set at 0.05, denoting a 5% risk of erroneously identifying a difference; and the type II error rate (β), established at 0.2, corresponding to an 80% statistical power to detect true effects. Furthermore, standard deviations for the serum biomarkers and the studied hormones, derived from existing literature [36–39], were incorporated into the analysis. These carefully selected inputs facilitated the accurate estimation of the sample size required to robustly identify significant variations in the levels of NEAT1, miR-34a, and the specified serum hormones.

### Sample collection

Blood samples were drawn from the antecubital vein following a minimum fasting period of 8 h. These samples were collected in yellow gel vacutainers and left undisturbed for 30 min. The vacutainers were centrifuged at 4000 rpm for 10 min to facilitate the separation of serum from the clot. The resulting serum aliquots were preserved at − 80 °C until the time of RNA extraction.

### RNA extractions

Total RNA was extracted from 200 µL of serum, ensuring no hemolysis, using miRNeasy extraction kits (Qiagen, Valencia, CA, USA) according to the manufacturer’s guidelines. The RNA’s concentration and purity were assessed with a NanoDrop2000 (Thermo Scientific, Waltham, MA, USA).

### Reverse transcription reactions

Total RNA was subjected to reverse transcription in a 20 µL reaction volume, consisting of 11 µL of RNA, 2 µL of genomic DNA elimination (GE) solution, and 7 µL of the reverse transcription mix. Utilizing the RvertAid First Strand cDNA Synthesis Kit (Thermo Fisher Scientific, USA), cDNA synthesis was carried out following the manufacturer’s instructions.

### Quantitative real-time polymerase chain reaction

The cDNA samples underwent amplification as per the manufacturer’s guidelines using the miScript SYBR Green PCR Kit (Qiagen), which included the miScript Universal Primer (reverse primer) and specific primers for miRNA-34a (forward primer). SNORD68, a component of the miScript PCR control miRNA, was used as the internal reference gene, consistent with previous reports [40]. For the RT2 lncRNA PCR assay focusing on NEAT1, the specified primer set (catalog no. 330,701 LPH15809A, Accession no. NR_028272.1) was utilized, with GAPDH serving as the internal housekeeping gene [41, 42] (catalog no. 330,701 LPH31725A, Accession no. ENST00000496049.0).

A 20 µL reaction mixture for the RT‒PCR was prepared, including 10 µL of master mix, 2 µL of the predesigned assay primer, 2.5 µL of diluted cDNA, and 5.5 µL of RNase-free water. The PCR settings involved an initial step at 95 °C for 10 min, followed by 45 cycles of 95 °C for 15 s and 60 °C for 60 s. Gene expression levels were assessed using the 2^−ΔCt^ method and normalized to the internal control. The specificity of the RT‒PCR products was confirmed via melt curve analysis. Fold changes were calculated using the 2^−ΔΔCt^ method for relative quantification [43]. with ΔΔCt for the control sample set to zero, making 2^0 equal to one [44].

### Bioinformatics approach for building coexpression networks of lncRNA, miRNA, and target genes

**Fig. 1:**
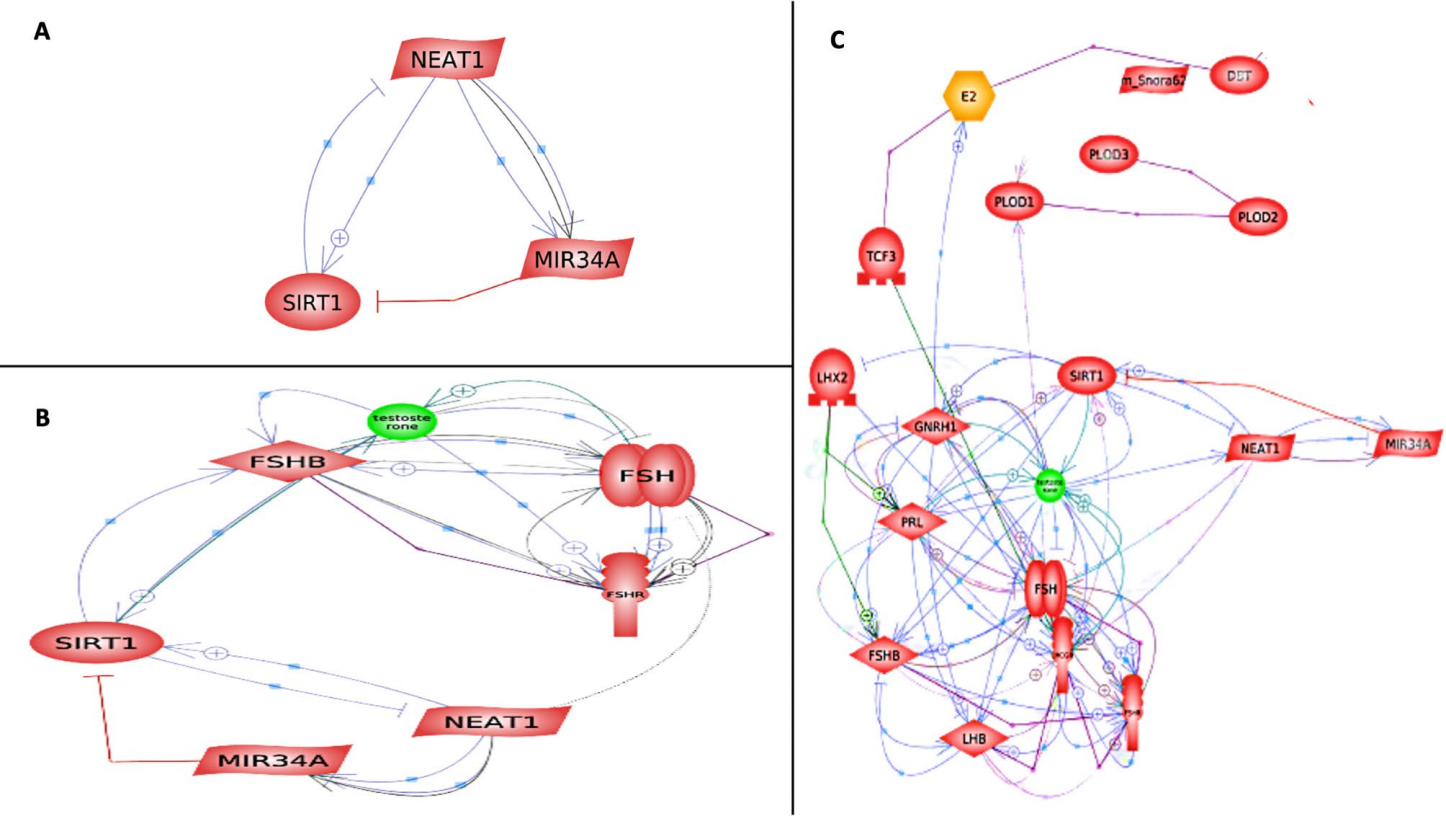
A comprehensive network and visualization tool illustrating the molecular interactions among the genes analyzed was created using the Pathway Studio online platform. NEAT1, miR-34a, FSH, total testosterone, prolactin, estrogen and Sirt1

The studied ncRNAs (NEAT1 and miR-34a) were tested with the hormonal panel FSH, total testosterone, prolactin, estrogen and the Sirt1 protein to test the hypothesis of biological network interaction using the Integrated Network and Visualization Tool “EmBiology-ELSEVIER Pathway Studio online tool”, and the findings revealed the molecular interactions among the genes examined, the hormones analyzed, and notably the relationship between NEAT1 and its downstream targets (Fig. 1).

To test the link between the target genes and the studied ncRNAs, a KEGG tool was used, which revealed the involvement of the targeted gene SIRT1 in the miR-34a signaling pathway in other studies and diseases such as prostate cancer (Supplementary Fig. 1). NEAT1 was identified as one of the top ncRNAs strongly associated with infertility based on data from the LncRNA and Disease Database, a partner of BIGD (https://www.cuilab.cn/lncrnadisease) (Supplementary Table 1). This database highlights NEAT1’s causal link to corpus luteum dysfunction, where low progesterone levels are a primary contributor to reduced fertility. We conducted an in-depth analysis of NEAT1’s potential miRNA targets utilizing the transcriptome-wide predictions from the miRcode 11 database (http://www.mircode.org/). Notably, the miR-34ac/34bc-5p and 449abc/449c-5p families emerged as significantly conserved targets of NEAT1. To further investigate the connection of these miRNAs with male infertility, we validated our findings through the Human MicroRNA Disease Database v4 (https://www.cuilab.cn/hmdd). Additionally, we utilized TargetScan 7.2 (https://www.targetscan.org/vert_72/) to identify potential targets for miR-34-5p/449-5p and miR-34a-3p, revealing SIRT1 as a key biological target of miR-34a-3p. The relationship between miR-34a and SIRT1 was subsequently confirmed via miRcode 11.

### Statistical analysis

Statistical evaluations were conducted using GraphPad Prism software (version 9.0, GraphPad Software, USA). The Shapiro-Wilk test was utilized to assess data normality. For normally distributed data, results were expressed as mean ± standard deviation (SD), and comparisons among groups were made using one-way ANOVA, complemented by Tukey’s post hoc test for multiple comparisons. In cases of non-normally distributed data, results were presented as median and interquartile range (IQR), with the Kruskal-Wallis test employed for comparisons, followed by Dunn’s post hoc test for detailed analysis. Categorical variables were assessed using the chi-square test.

To evaluate the diagnostic effectiveness of the biomarkers studied, receiver operating characteristic (ROC) curve analysis was performed. The area under the curve (AUC) was calculated to determine the sensitivity, specificity, and accuracy of the biomarkers in distinguishing between groups. Optimal cut-off points were established based on the Youden index. Correlations among variables were analyzed using Pearson or Spearman correlation coefficients, depending on the data’s distribution. A significance level was set at *p* < 0.05. Data visualization included graphical representations such as box plots and ROC curves to illustrate data distribution and diagnostic performance.

## Results

### Demographic analysis

**Fig. 2:**
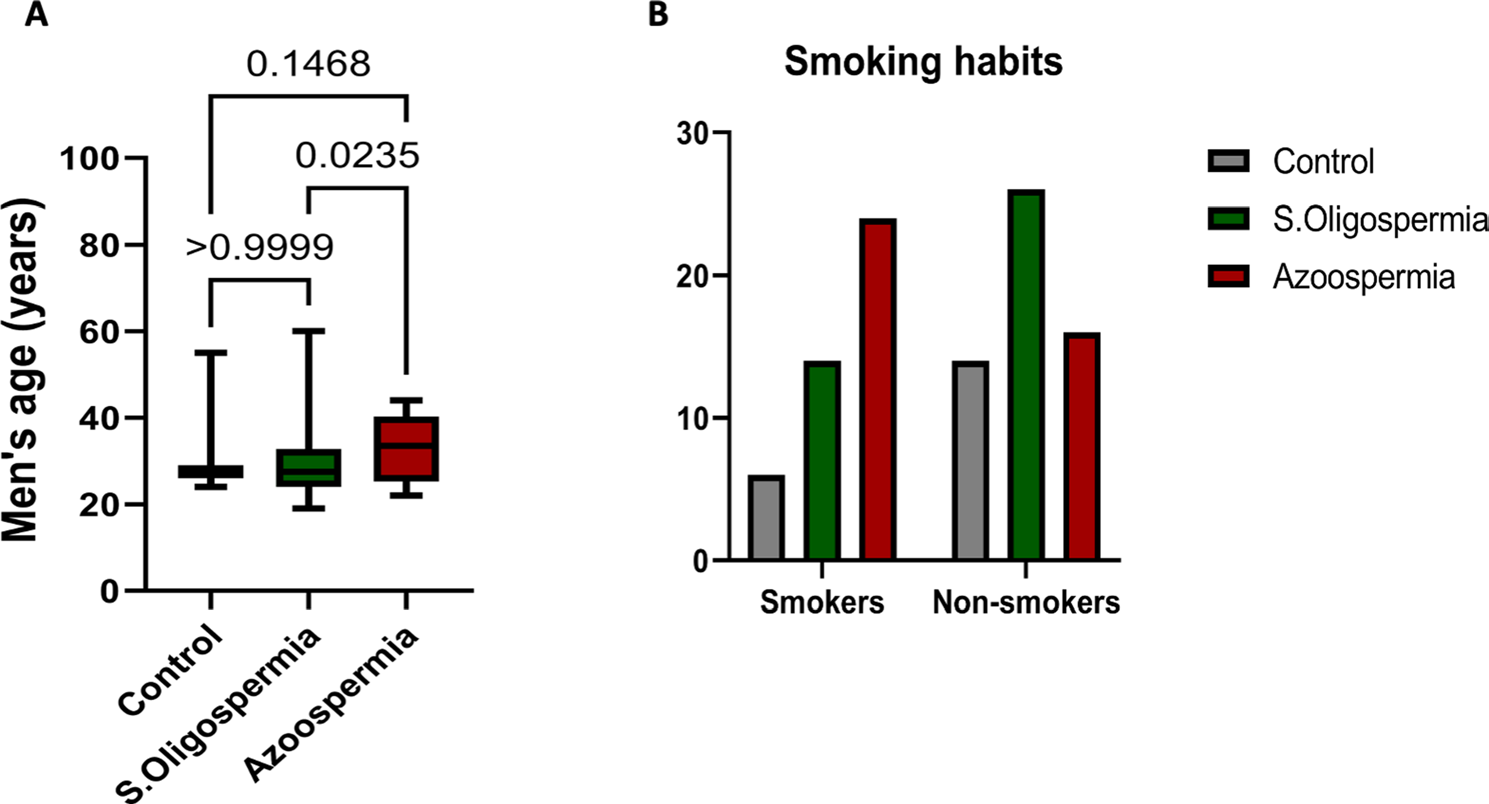
Age (**A**) and smoking habit (**B**) were compared between men with severe oligospermia and azoospermia patients and controls. Age data was illustrated using a box plot, where the box indicates the interquartile range (25th to 75th percentiles). The median is represented by a line within the box, while the upper and lower whiskers denote the 10th to 90th percentiles. Additionally, smoking behaviors were analyzed using the chi-square test, with a significance level set at *p* < 0.05

The BMI values for the non-obstructive azoospermia group (28.2 ± 5.4 kg/m²), severe oligospermia group (26.4 ± 6.4 kg/m²), and control group (27.3 ± 4.9 kg/m²) showed no significant difference between the studied groups (*p* = 0.722). The ages of patients diagnosed with non-obstructive azoospermia and severe oligospermia were comparable to those in the control group. However, within the patient groups, the average age of individuals with non-obstructive azoospermia was significantly higher than that of those with severe oligospermia (*p* = 0.0235) (Fig. 2A). Smoking habits were assessed by directly asking the patients about their smoking status. There were no significant variations in smoking habits among the different groups (Fig. 2B).

### Serum expression levels of NEAT1 and miR-34a in patients with severe oligospermia and non-obstructive azoospermia

**Fig. 3:**
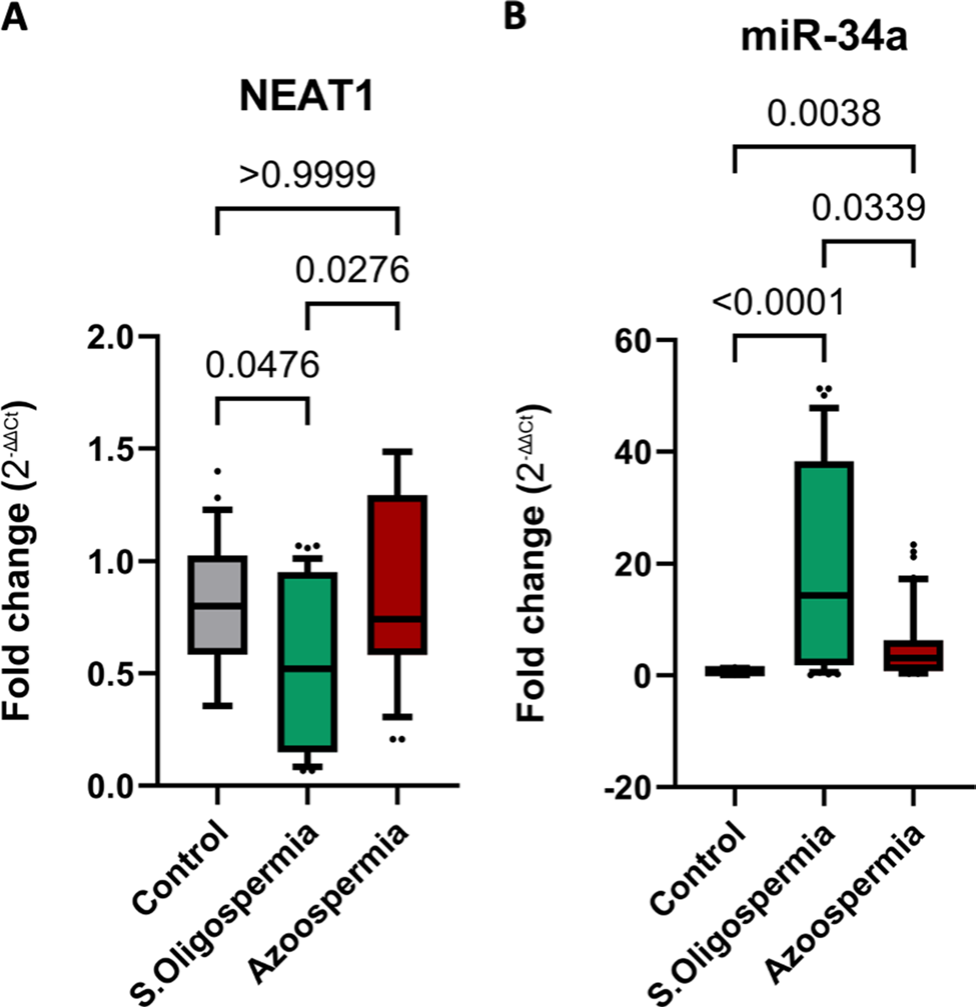
The expression levels of serum NEAT1 and miR-34a were analyzed in patients with severe oligospermia (*n* = 40) and non-obstructive azoospermia (*n* = 40) relative to healthy controls (*n* = 20). The 2^−∆∆Ct^ values for the control group were calculated by taking the average control value and subtracting each individual control measurement. The data is presented as box plots, where the boxes indicate the interquartile range (from the 25th to the 75th percentile), the lines within the boxes represent the medians, and the whiskers extend to the 10th and 90th percentiles. Statistical significance was set at *p* < 0.05

In patients with severe oligospermia, serum NEAT1 levels were significantly lower than in healthy controls, with a median fold change of 0.52 (IQR: 0.15–0.95; *p* = 0.0476). In contrast, patients with non-obstructive azoospermia did not show a significant difference, displaying a median fold change of 0.74 (IQR: 0.58–1.29; *p* > 0.9999). Additionally, NEAT1 levels were significantly reduced in those with severe oligospermia compared to non-obstructive azoospermia patients (*p* = 0.0276) (see Fig. 3A).

On the other hand, serum levels of miR-34a were significantly increased in both severe oligospermia and non-obstructive azoospermia patients compared to healthy controls, with median fold changes of 2.86 (IQR: 1.032–19.83; *p* < 0.0001) and 3.82 (IQR: 1.633–14.45; *p* = 0.0038), respectively. Furthermore, miR-34a levels were significantly higher in severe oligospermia patients compared to those with non-obstructive azoospermia (*p* = 0.0339) (see Fig. 3B).

### Serum hormone levels

**Fig. 4:**
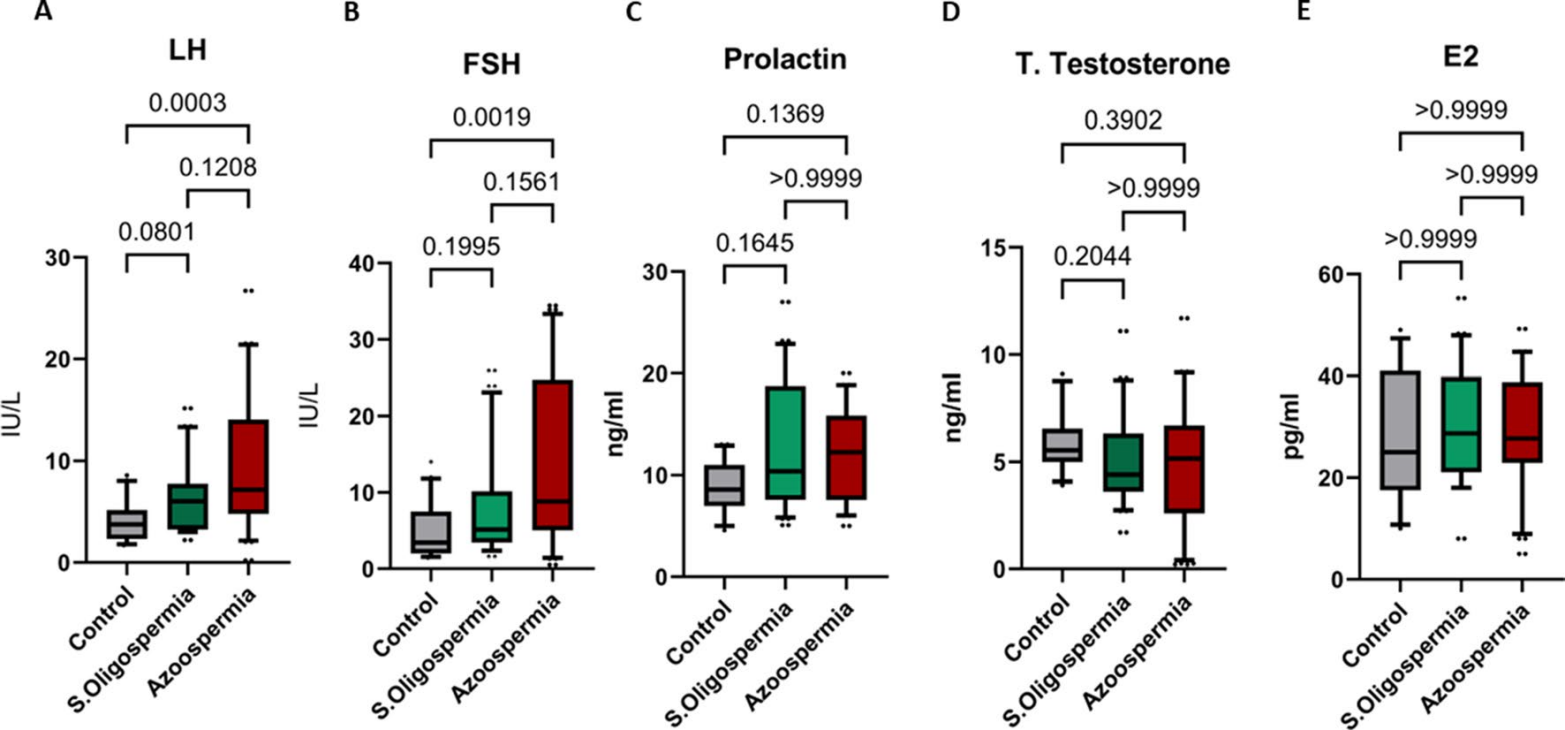
Serum concentrations of **(A)** LH, **(B)** FSH, **(C)** prolactin, **(D)** testosterone, and **(E)** estradiol in individuals with severe oligospermia (*n* = 40) and non-obstructive azoospermia (*n* = 40) compared to healthy controls (*n* = 20). Data are displayed as box plots, where the boxes illustrate the interquartile range (25th to 75th percentiles), the horizontal lines within the boxes indicate the median values, and the whiskers represent the 10th to 90th percentiles. A p-value of less than 0.05 denotes statistical significance

The serum levels of LH, FSH, prolactin, testosterone, and E2 are shown in (Fig. 4**)**. The serum LH and FSH concentrations were considerably greater in the non-obstructive azoospermia group than in the control group, with medians (IQRs) of 7.15 (4.8–14.08) (*p* = 0.0003) and 8.8 (5.05–24.75) (*p* = 0.0019), respectively. The concentrations of prolactin, total testosterone, and estradiol differ between the groups but do not appear to do so significantly. However, it indicates that patient groups are slightly higher compared to control, although the difference is not much it shows an increasing trend of prolactin levels. Likewise, the serum total testosterone is marginally lower in the patient groups than it is in the control, suggesting a decline in this parameter. The estradiol on the other hand exhibits fluctuating levels between the groups and no general emphasis on increasing or decreasing. While these observations are not significant, they raise the possibility of changes to these hormonal markers.

### Correlations between the studied parameters and clinical features

**Fig. 5:**
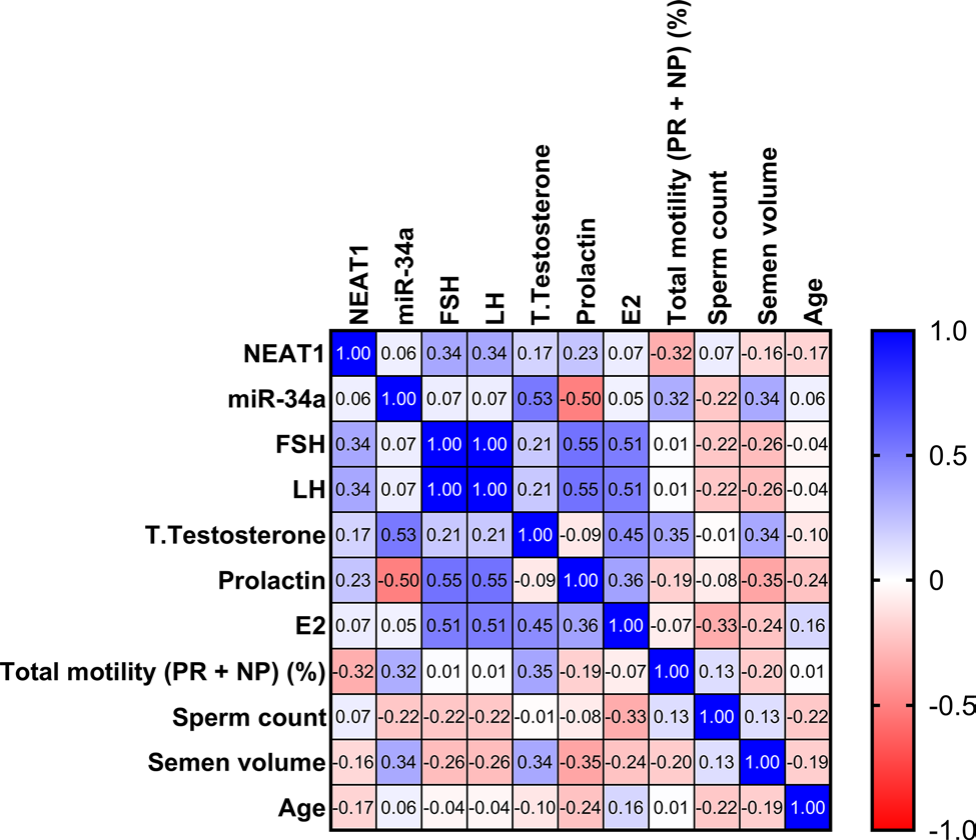
Relationships between the serum markers examined and their connection to clinical data in the group with severe oligospermia. The correlation map employs a blue-to-red gradient, where blue indicates a correlation approaching 1, and red signifies a correlation approaching − 1. Areas shown in white represent correlations close to 0. Spearman correlation analysis was utilized to evaluate these relationships

In the severe oligospermia group, we noted a significant positive correlation between NEAT1 expression and levels of LH and FSH (*r* = 0.34, *p* = 0.04). Furthermore, a notable positive relationship was observed between miR-34a expression and total sperm motility (*r* = 0.32, *p* = 0.044). Conversely, an inverse correlation was identified between miR-34a expression and sperm count (*r* = − 0.22, *p* = 0.034), as illustrated in (Fig. 5**)**.

**Fig. 6:**
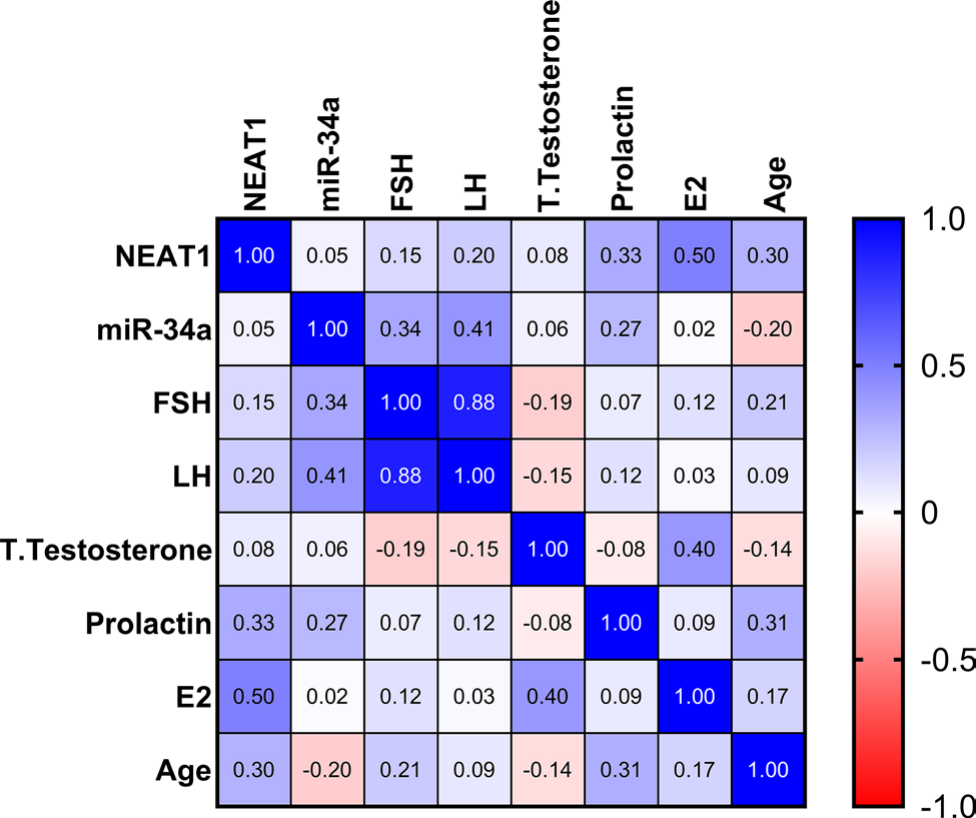
Relationships between the serum markers and clinical data within the non-obstructive azoospermia group. The correlation map uses a blue-red gradient, where blue represents a strong positive correlation (approaching 1), red indicates a strong negative correlation (nearing − 1), and white signifies little to no correlation (close to 0). To evaluate these associations, Spearman correlation analysis was utilized

In the azoospermia cohort, a significant positive relationship was observed between the expression of miR-34a and the concentrations of LH (*r* = 0.41, *p* = 0.007) and FSH (*r* = 0.34, *p* = 0.03), as shown in (Fig. 6).

### Diagnostic performance of the NEAT1 and miR-34a among studied groups

**Fig. 7:**
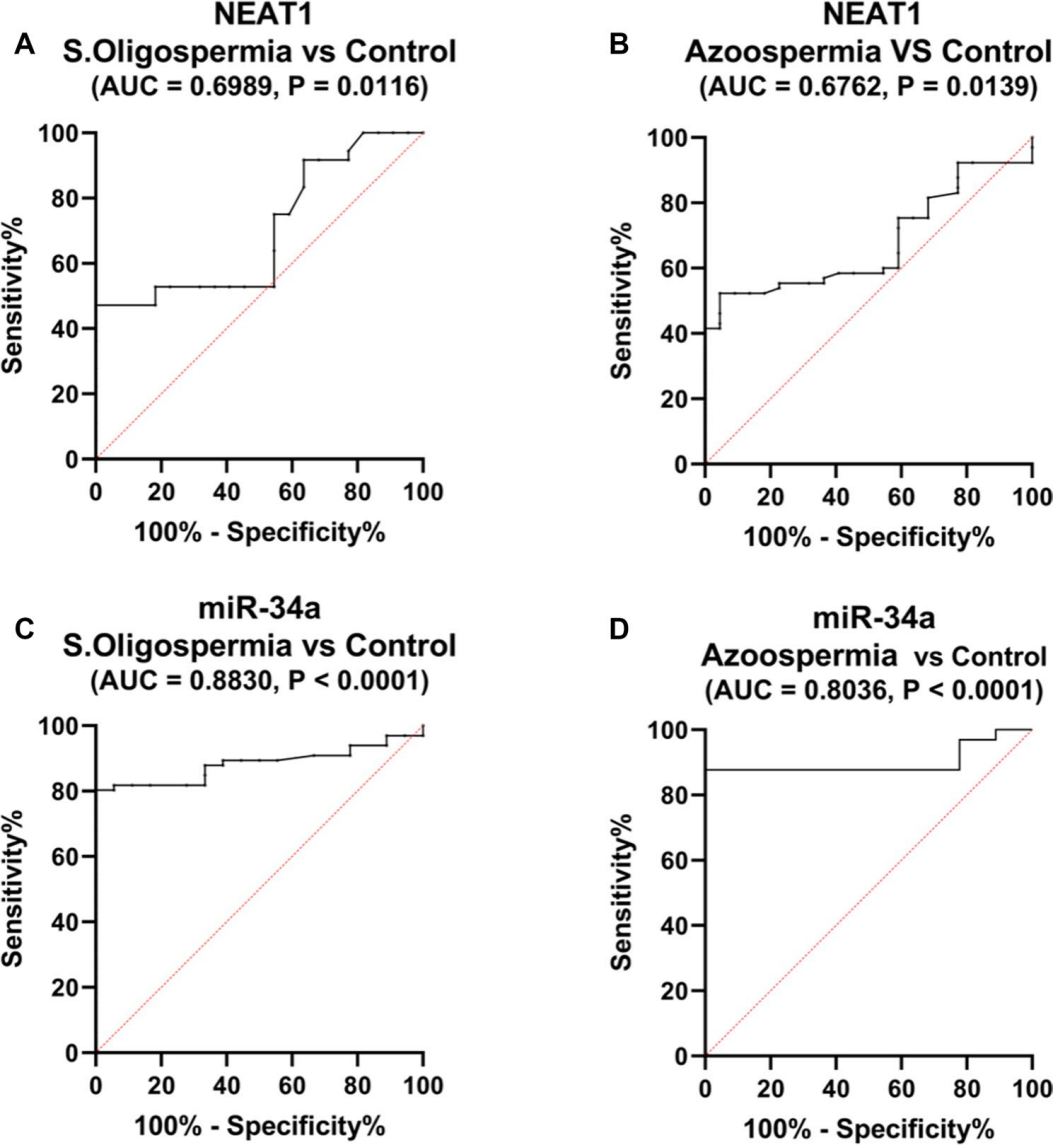
Evaluation of diagnostic performance of serum levels of **(A**,** B)** NEAT1 and **(C**,** D)** miR-34a. ROC curve analysis was performed to assess patients with severe oligospermia (*n* = 40), non-obstructive azoospermia (*n* = 40), and a control group (*n* = 20)

ROC curve analysis revealed that serum levels of NEAT1 can effectively differentiate patients with severe oligospermia from healthy individuals, achieving an AUC of 0.6989 (95% CI: 0.6148 to 0.9269, *p* = 0.01), with a sensitivity of 53% and specificity of 82% at a threshold greater than 0.53-fold (Fig. 7A).

In the case of non-obstructive azoospermia, serum NEAT1 levels also successfully distinguished these patients from healthy controls, yielding an AUC of 0.6762 (95% CI: 0.7820 to 0.9977, *p* = 0.01), with 52% sensitivity and 95% specificity at a cutoff above 1.29 **(**Fig. 7B**).**

For miRNAs, serum miR-34a levels were shown to differentiate severe oligospermia patients from healthy controls, presenting an AUC of 0.883 (95% CI: 0.7424 to 0.9972, *p* < 0.0001), demonstrating a sensitivity of 80% and specificity of 94% at a threshold exceeding 1.37-fold (Fig. 7C).

Additionally, miR-34a levels were effective in distinguishing non-obstructive azoospermia patients from healthy controls, with an AUC of 0.8036 (95% CI: 0.7424 to 0.9972, *p* < 0.0001), achieving a sensitivity of 87.69% and specificity of 94.44% at a cutoff greater than 1.34-fold (Fig. 7D).

### Diagnostic performance of LH and FSH between the studied groups

**Fig. 8:**
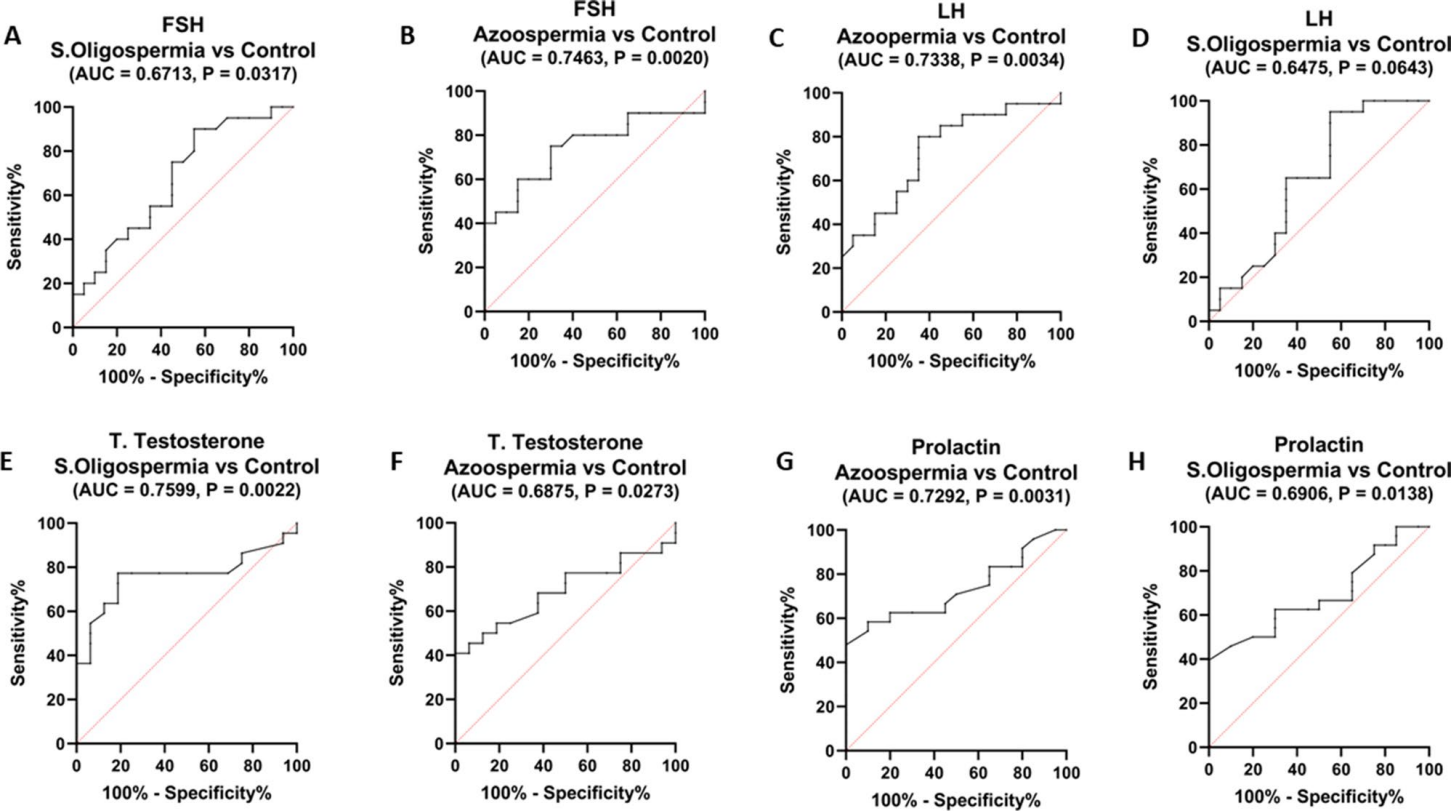
Assessment of diagnostic performance for serum concentrations of **(A**,** B)** FSH, **(C**,** D)** LH, **(E**,** F)** total testosterone, and **(G**,** H)** prolactin levels through ROC curve analysis in cases of severe oligospermia (*n* = 40), non-obstructive azoospermia (*n* = 40), and control subjects (*n* = 20)

In a ROC analysis evaluating hormonal levels, patients with severe oligospermia were differentiated from healthy controls, yielding an AUC of 0.6713 (95% CI: 0.5224 to 0.8201, *p* = 0.03). This analysis indicated a sensitivity of 90% and a specificity of 45% with a threshold of > 3.00-fold for FSH. For LH, the AUC was 0.7338 (95% CI: 0.483 to 0.812, *p* = 0.0643), achieving a sensitivity of 95% and a specificity of 45% at a cutoff > 2.95-fold (Fig. 8A, C).

Furthermore, both total testosterone and prolactin levels effectively distinguished severe oligospermia from healthy individuals. Testosterone presented an AUC of 0.7599 (95% CI: 0.638 to 0.8819, *p* = 0.002), with a sensitivity of 77.27% and specificity of 81.25% at a threshold of < 5.23-fold. Prolactin had an AUC of 0.6906 (95% CI: 0.5646 to 0.8166, *p* = 0.0138), demonstrating a sensitivity of 62.5% and specificity of 70% with a cutoff > 10.10-fold (Fig. 8E, G).

For distinguishing non-obstructive azoospermia from healthy controls, FSH showed an AUC of 0.7463 (95% CI: 0.6227 to 0.8698, *p* = 0.002), with sensitivity at 75% and specificity at 70% for a cutoff > 6.65. LH had an AUC of 0.7338 (95% CI: 0.6010 to 0.8665, *p* = 0.034), revealing 80% sensitivity and 65% specificity at a cutoff > 4.6 (Fig. 8B, D).

Additionally, testosterone’s AUC was 0.6875 (95% CI: 0.5562 to 0.8188, *p* = 0.03), with both sensitivity and specificity at 95.45% for a cutoff > 10.45-fold. Prolactin had an AUC of 0.7292 (95% CI: 0.6122 to 0.8461, *p* = 0.01), achieving a sensitivity of 62.5% and specificity of 80% at a cutoff > 11.45-fold (Fig. 8F, H).

## Discussion

Male infertility can arise from altered spermatogenesis, testicular disorders, hormonal imbalances, or sperm dysfunction [45–47]. Quantitatively, these sperm production disorders manifest as oligospermia (low sperm count) or non-obstructive azoospermia (absence of sperm during ejaculation). Research has linked various genes to spermatogenesis, and there is increasing interest in the role of lncRNAs and miRNAs in diagnosing and treating male infertility [47–49]. However, the relationship between miRNA expression patterns in serum and male infertility remains poorly understood. This study assessed the expression levels of ncRNAs NEAT1 and miR-34a in the serum of patients with non-obstructive azoospermia and severe oligospermia compared to those with normal fertility.

The hypothalamic-pituitary-gonadal (HPGA) axis plays a crucial role in spermatogenesis. At puberty, the hypothalamus secretes gonadotropin-releasing hormone (GnRH), stimulating the anterior pituitary gland to release FSH and LH. FSH supports Sertoli cells in sperm production, while LH prompts Leydig cells to produce testosterone, crucial for spermatogenesis. Defects in any component of the HPGA can lead to impaired spermatogenesis, including non-obstructive azoospermia [50].

Sertoli and Leydig cells play essential roles in spermatogenesis; Leydig cells produce testosterone, and Sertoli cells support germ cell maturation [47, 51]. Non-obstructive azoospermia typically results from inadequate gonadotropin stimulation or intrinsic testicular disorders [52]. Hypogonadotropic hypogonadism features low serum testosterone due to reduced LH and FSH release, while hypergonadotropic hypogonadism involves desensitized Sertoli cell receptors from elevated FSH levels [53, 54]. FSH levels are inversely correlated with spermatogonia numbers; higher FSH correlates with lower spermatogonia counts [55, 56]. The significantly higher serum FSH concentrations observed in the non-obstructive azoospermia group implies that there is abnormality in the end organ responsiveness to gonadotropin stimulation, a hallmark of this condition. This hormonal disturbance is further supported by the strong association between elevated FSH levels and spermatogenesis abnormalities. These observations underscore the role of hypogonadism in the differential analysis of male infertility, which, together with serum NEAT1 and miR-34a, has diagnostic significance.

Individuals with testicular non-obstructive azoospermia generally exhibit low testosterone and elevated FSH levels [51]. In cases of bilateral testicular atrophy, high FSH and low testosterone indicate primary testicular failure [57]. Total testosterone measurements combined with serum LH and FSH can aid in diagnosis [58, 59]. Serum concentrations of FSH and testosterone give essential data about testicular function and etiology of the infertility. According to abnormalities in these hormones, it is possible to distinguish between primary testicular failure and secondary causes of male infertility, which in turn can lead to more specific diagnoses. The integration of serum NEAT1 and miR-34a in diagnostic approaches could be beneficial and extend the likelihood of identifying concrete causes of male infertility even more.

The significant positive correlation between NEAT1 expression and gonadotropin levels in severe oligospermia suggests that NEAT1 may enhance the hormonal environment supporting spermatogenesis [60]. The positive correlation between miR-34a expression and gonadotropin levels highlights its regulatory role in feedback mechanisms.

Spermatogenesis cannot progress without testosterone. Patients with non-obstructive azoospermia have lower testosterone levels than fertile males, with non-obstructive azoospermia patients exhibiting significantly lower levels than obstructive ones [61]. Liu et al. [62]. reported that non-obstructive azoospermia patients had significantly lower testosterone levels than fertile males. This observation is in line with our study since the testosterone level was observed to be lower in the severe oligospermia and non-obstructive azoospermia groups compared to control subjects. Since testosterone is required for spermatogenesis, our findings underline the role of the hormone in male infertility. And when integrated with NEAT1 and miR-34a biomarker, these findings may improve diagnostic accuracy and contribute to the development of treatment approaches for male infertility.

Estrogens, particularly estradiol (E2), synthesized from testosterone, are crucial for libido and spermatogenesis [63]. Subfertile and non-obstructive azoospermic individuals may have abnormal estrogen levels. Hargreave et al. [64]. reported that subfertile patients had lower blood E2 levels than fertile males. Salama and Blgozah [65] reported a wide spectrum of serum E2 concentrations in patients with non-obstructive azoospermia and fertile men.

Changes in E2 levels, whether high [66] or low [67, 68], may be related to impaired spermatogenesis, as evidenced by clinical investigations. A high E2 level may impact Leydig cell activity by directly decreasing Leydig cell function and testosterone production [68], as well as inhibiting pituitary gonadotrophin secretion [69]. Reduced E2 levels, on the other hand, might affect estrogen signaling pathways, which have been linked to germ cell development [70]. The testosterone/E2 ratio may serve as a diagnostic indicator in these patients [71].

Despite no significant differences in mean estradiol being found between groups, the different patterns depicted in the results could imply changes to hormonal balance. These changes may slightly affect spermatogenesis as earlier research shows that low or high levels of estradiol affect spermatogenesis.

Prolactin, produced in the anterior pituitary, can disrupt steroidogenesis and spermatogenesis via its receptors in Sertoli and Leydig cells [72].

Previous research has shown a significant inverse link between prolactin and sperm effectiveness. According to previous studies, blood prolactin levels are substantially greater in non-obstructive azoospermic patients than in healthy males [73, 74]. Schoor et al. reported that blood prolactin levels were greater in non-obstructive azoospermic patients than in obstructive azoospermia patients [75]. Ellithy et al. reported that non-obstructive azoospermic patients had significantly greater blood prolactin levels than normozoospermic males [76].

Our findings are consistent with previous studies, which show elevated levels of FSH and LH and decreased testosterone levels in the non-obstructive azoospermia group. Furthermore, prolactin levels were significantly higher in these patients compared to the control group.

The miR-34 family of miRNAs has been hypothesized to be key modulators of the p53 pathway and possible tumor suppressors in human malignancies [77]. The differentiation and proliferation of a range of different cell types are regulated by miR-34a. MiR-34a was previously identified as a tumor suppressor gene [78–80]. Numerous miRNAs have been found in spermatozoa, and they may serve as important apoptotic controllers. Since spermatogenesis is tightly regulated by apoptosis, alterations in miRNA expression could be a contributing factor to male infertility [81–83]. Consequently, alterations in miRNA expression may be a factor in male infertility.

MiR-34a is a member of the miR-34 family that is highly expressed in testicles. The miR-34 family includes miR-34a, miR-34b, and miR-34c. MiR-34b and miR-34c play important roles in infertility and have been linked to spermatogenesis, sperm function, fertilization, and early embryonic development, according to previous studies [84–86]. Earlier reports have shown that miR-34a has a regulatory role in spermatocyte meiosis and spermiogenesis, where it is upregulated in mature mouse testes, induces apoptosis and inhibits proliferation [79, 87].

In a study of individuals with severe oligospermia revealed that the expression of miR-34a was significantly upregulated in these patients compared to fertile control individuals and those with non-obstructive azoospermia. Additionally, miR-34a expression was upregulated in individuals with non-obstructive azoospermia compared to fertile individuals. Concerning diagnostic accuracy, miR-34a showed good diagnostic accuracy in the severe oligospermia group as well as the non-obstructive azoospermia group compared to the control fertile group. MiR-34a has been identified as a pro-apoptotic microRNA in a variety of cell types [11]. Previously, it was reported that miR-34a overexpression might influence sperm motility in zebrafish [10]. A number of mRNAs, including apoptotic (p53) and antiapoptotic (Bcl2 and Sirt1) genes, have been identified as miR-34a targets [85, 88]. The putative binding site of miR-34a is located in the 3’UTR of SIRT1 [78]. The transcriptional regulation of Sirt1 expression by miR-34a has been demonstrated.

Sirt1 is a class III protein deacetylase that can affect numerous transcription factors, including those in charge of controlling reactive oxygen species (ROS) production, such as FoxO pathways, and can modify DNA activity by histone deacetylation [10]. It appears that oxidative stress and higher amounts of ROS, in turn, can influence Sirt1 activity [89]. Jiao et al. reported that the miR-34a/Sirt-1 pathway plays a significant role in diabetes-induced testicular apoptotic cell death, suggesting that miR-34a inhibition and SIRT1 activation could be used as innovative therapeutic methods for treating diabetes mellitus-induced male infertility [17]. Thus, SIRT1 may be a miR-34a target that helps to support apoptosis.

The results of our study which revealed an increase in miR-34a expression in both the severe oligospermia and non-obstructive azoospermia patients support the literature that has described miR-34a as a pro-apoptotic molecule. This implies that miR-34a could be involved in sperm dysfunction through control of the apoptotic signals. Furthermore, the high diagnostic accuracy of miR-34a demonstrated in the present study further substantiates its application as a biomarker for male infertility in severe oligospermia and non-obstructive azoospermia, as well as the possibility of the role of miR-34a via pathways such as the miR-34a/SIRT1 pathway in these conditions.

Not only are miRNAs of interest, but lncRNAs are also a current area of research. Long ncRNAs are among the main types of ncRNAs. LncRNAs play a range of roles in biological processes, such as cell differentiation, proliferation, death, and the clinical manifestations of numerous diseases. In 2017, scientists discovered that 1,534 lncRNAs were differentially expressed in round sperm cells and pachytene spermatocytes, while 1,630 lncRNAs were differentially expressed in spermatogonia [23]. The greater abundance of lncRNAs expressed in the testis than in other tissues suggests that these lncRNAs may be crucial for male fertility [90]. Recent research has demonstrated the significance of lncRNAs in the growth, differentiation, and death of spermatogonia [91].

NEAT1 is a well-characterized lncRNA that has been substantially conserved throughout evolution and is widely expressed in mammalian cells. NEAT1 is involved in various activities, such as cell differentiation, inflammation, and stress response [92, 93]. NEAT1 is located in the nucleosome and is involved in spermatogenesis [94]. However, research on the association between the lncRNA NEAT1 and non-obstructive azoospermia is limited.

We evaluated NEAT1 expression in both non-obstructive azoospermia and severe oligospermia groups. The data showed significant downregulation of NEAT1 in the severe oligospermia group compared to the fertile male group. However, in the non-obstructive azoospermia group, NEAT1 was downregulated, but the difference was not statistically significant compared to the fertile male group results. Nkagawa et al. reported that reduced sperm quality and low fertility are linked to decreased expression of NEAT1 in mice [94]. Regarding the diagnostic accuracy of the markers under investigation, circulating miRNAs and lncRNAs are readily available, reliable, and accurate genetic tests for various diseases, such as non-obstructive azoospermia and severe oligospermia [95, 96]. In this study, we found that serum NEAT1 and miR-34a were differentially expressed between individuals with severe oligospermia and individuals with non-obstructive azoospermia and healthy controls, indicating that serum NEAT1 and miR-34a are potential novel biomarkers for non-obstructive azoospermia and severe oligospermia diagnosis. Notably, miR-34a showed greater accuracy, sensitivity and specificity than did NEAT1. These findings indicate that serum NEAT1 and miR-34a are reliable noninvasive early biomarkers and promising therapeutic targets for non-obstructive azoospermia and severe oligospermia treatment. Although the combination of NEAT1 and miR-34a with other biomarkers, such as specific hormones, may aid in non-obstructive azoospermia and severe oligospermia diagnosis, this needs further investigation.

The diagnostic evaluation of the hormones studied demonstrated significant sensitivity and specificity, suggesting the potential of FSH, LH, testosterone, and prolactin in distinguishing between healthy individuals and patients with non-obstructive azoospermia, as well as those with severe oligospermia. These results collectively indicate that using these hormones as a combined biomarker profile could improve the accuracy of diagnosis and differentiation between the specified patient groups and healthy controls.

There is a variation in lncRNA expression between men with non-obstructive azoospermia and healthy counterparts. LncRNAs can act as host genes for miRNAs, indirectly regulating target protein expression and consequently influencing spermatogenesis [97]. Furthermore, by binding to miRNAs at competitive sites, lncRNAs may act as ceRNAs, modulating spermatogenesis and maturation by reducing miRNA levels and diminishing their activity [28, 97]. The potential regulatory relationship between NEAT1 and miR-34a was also explored, revealing a negative correlation. Reduced NEAT1 expression led to increased miR-34a levels.

However, evidence for interactions between miRNAs and lncRNAs is becoming increasingly abundant. Previously reported that retinoblastoma invasion and proliferation can be inhibited by the miR-148b-3p/ROCK1 axis via the role of the lncRNA NEAT1 [98]. Moreover, NEAT1 upregulation controls ZEB1 expression via miR-194 to help ovarian cancer cells resist paclitaxel through drug resistance. Instead, Fu et al. confirmed that NEAT1 downregulation enhances the sensitivity of gemcitabine-resistant pancreatic cancer cells to gemcitabine via the miR-506-3p/ZEB2/EMT axis [99]. Furthermore, in osteoarthritic chondrocytes, miR-150-5p expression was elevated when NEAT1 was knocked down [100]. In non-small cell lung cancer, through the sponging of miR-153-3p, NEAT1 downregulation could inhibit non-small cell lung cancer cell proliferation, migration, and invasion while encouraging cell death [101].

Interestingly, a recent study showed that NEAT1 downregulation led to upregulated expression of miR-34a in cervical cancer tissues. The immediate target of miR-34a, LDHA, is derepressed by NEAT1, which serves as a ceRNA to sponge miR-34a [31]. In theory, our results are in line with those of previous reports. Subsequently, with the aid of online bioinformatics tools, we discovered that miR-34a, which serves as a connecting carrier, targets both NEAT1 and the 3’-UTR of SIRT1, consistent with the findings of Luo et al., who provided the first proof that a signaling axis involving NEAT1-miR-34a/SIRT1-Wnt/-catenin exists and that it may be exploited to treat colorectal cancer [33]. Our results provide preliminary evidence regarding the potential role of the NEAT1/miR-34a/SIRT1 axis, highlighting its possible relevance in understanding the molecular mechanisms underlying non-obstructive azoospermia and oligozoospermia. However, further studies with larger cohorts are required to validate these findings and explore their therapeutic implications.

However, it is important to recognize definite limitations that constrain the results of this study. First, the use of suitable cell lines is crucial for validating the ncRNA-related axis that were analyzed. Second, future clinical studies should consider including a larger cohort. Additionally, assessing SIRT1 in future research will be essential to further explore this hypothesis. Lastly, the parameters examined in this study should also be analyzed in semen samples.

## Conclusion

This study highlights the diagnostic potential of serum NEAT1 and miR-34a for non-obstructive azoospermia and severe oligospermia. These noninvasive biomarkers offer insights into the etiology of male infertility and may pave the way for personalized therapeutic approaches. Further investigations, including hormonal profile evaluation and combined biomarker analysis, are warranted to improve the diagnostic accuracy and treatment efficacy for male infertility.

## Electronic supplementary material

Below is the link to the electronic supplementary material.

Supplementary Material 1

## Data Availability

No datasets were generated or analysed during the current study.
