# Supplementary material for: A comparison of the expression patterns and diagnostic capability of the ncRNAs NEAT1 and miR-34a in non-obstructive azoospermia and severe oligospermia
Source: Hum Genomics. 2025 Mar 31;19:35. doi: 10.1186/s40246-025-00742-9 (PMC11959825; doi:10.1186/s40246-025-00742-9)
Supplement: Supplementary file 1 — Supplementary Material 1 [file 40246_2025_742_MOESM1_ESM.docx]

##### **
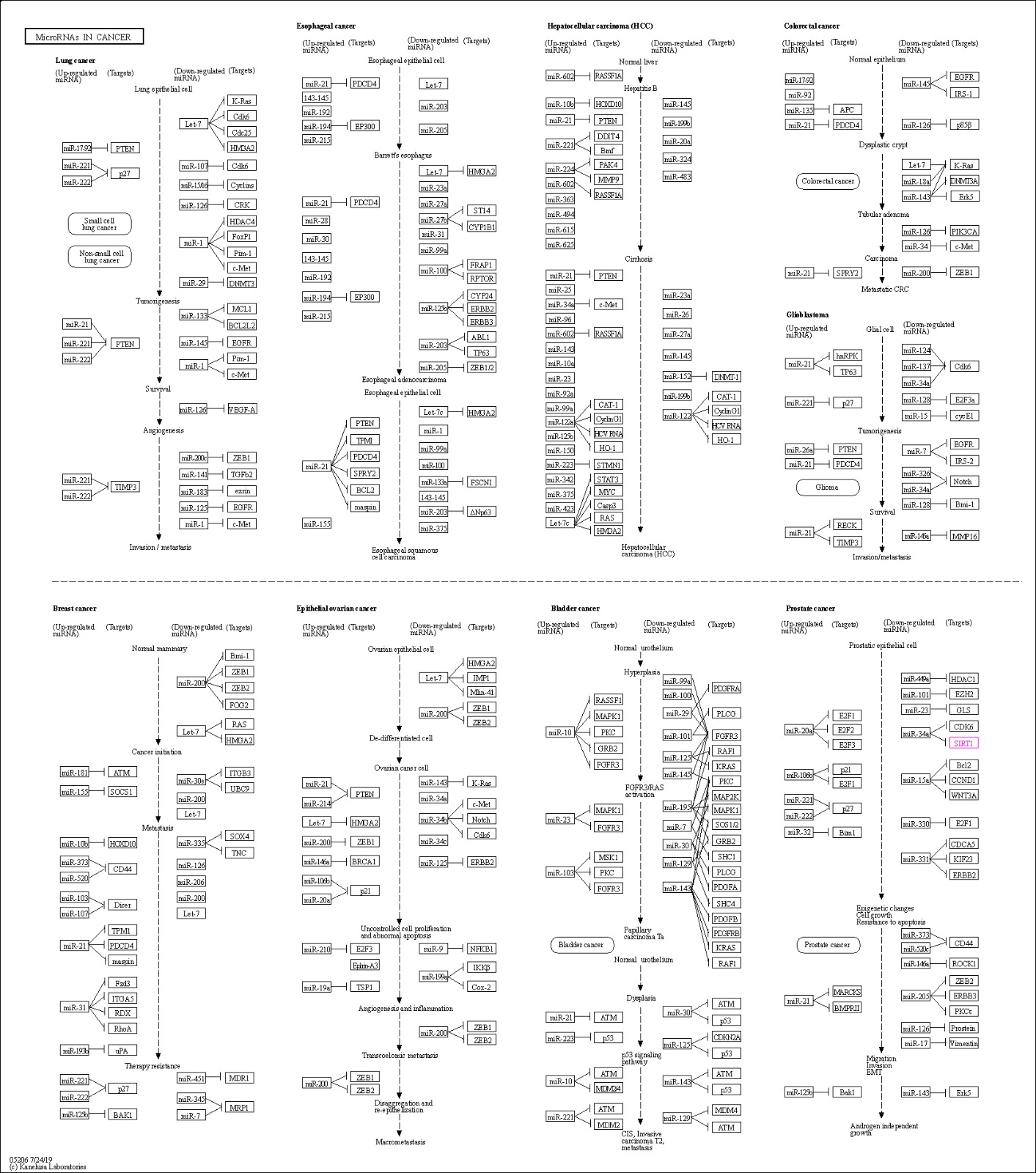
Supplementary Figure 1:** KEGG diagram showing the involvement of the targeted gene SIRT1 in combination with the miR-34a signaling pathway in other studies and diseases such as prostate cancer. The gene of interest is colored purple.

| **LncRNA name** | **Disease name** | **Dysfunction type** | **Description** | **Chr** | **Species** | **Alias** | **GenBank** | **Sequence** | **Reference** |
| --- | --- | --- | --- | --- | --- | --- | --- | --- | --- |
| NEAT1 | infertility | Expression | Neat1 knockout (KO) mice stochastically fail to become pregnant despite normal ovulation. Unilateral transplantation of wild-type ovaries or the administration of progesterone partially rescued the phenotype, suggesting that corpus luteum dysfunction and concomitant low progesterone were the primary causes of the decreased fertility. In contrast to the faint expression observed in most of the adult tissues, Neat1 was highly expressed in the corpus luteum, and the formation of luteal tissue was severely impaired in nearly half of the Neat1 KO mice | chr11 | Human | LINC00084; NCRNA00084; TncRNA; VINC | NR_131012 | Gene / RNA | 25359727 |

**Supplementary Table 1.** LncRNADisease: a database for long-noncoding RNA-associated diseases.
